# Carotenoid Profile in Maternal/Cord Plasma and Changes in Breast Milk along Lactation and Its Association with Dietary Intake: A Longitudinal Study in a Coastal City in Southern China

**DOI:** 10.3390/nu14091989

**Published:** 2022-05-09

**Authors:** Xinyao Dai, Huanhuan Yin, Jing Zhang, Fang Tian, Xiaokun Cai, Yingyi Mao, Hanxiao Sun, He Wang, Xiang Li, Hui-Lian Zhu, Lishi Zhang, Jinyao Chen, Yanrong Zhao

**Affiliations:** 1 West China School of Public Health and West China Fourth Hospital, Sichuan University, Chengdu 610041, China; 18328155522@163.com (X.D.); yinhuanhuan1998@163.com (H.Y.); zzbher@163.com (J.Z.); sunhanxiao875@163.com (H.S.); hh573135021@163.com (H.W.); lishizhang_56@163.com (L.Z.); 2 Abbott Nutrition Research & Development Centre, Shanghai 200233, China; fang.tian@abbott.com (F.T.); xiaokun.cai@abbott.com (X.C.); yingyi.mao@abbott.com (Y.M.); xiang.li@abbott.com (X.L.); 3 Department of Nutrition, School of Public Health, Sun Yat-sen University, Guangzhou 510080, China; zhuhl@mail.sysu.edu.cn

**Keywords:** human milk, carotenoids, lactation stage, lutein, maternal/cord plasma, China

## Abstract

In this study, changes of carotenoids in breast milk were observed longitudinally for up to one year. Our study aimed to analyze the profile of carotenoids in breast milk and maternal/cord plasma and its correlation with dietary intake in Guangzhou. Plasma and breast milk samples of five stages during lactation (i.e., colostrum; transitional milk; and early, medium, and late mature milk) were collected from lactating mothers. The food frequency questionnaire (FFQ) was used for collecting data on dietary intake in the corresponding stages. Levels of lutein, zeaxanthin, β-cryptoxanthin, β-carotene, and lycopene were analyzed by high-performance liquid chromatography. We found that the total carotenoid level decreased gradually with the extension of lactation and eventually stabilized. Among them, the content of lutein increased from colostrum to transitional milk and decreased thereafter until it plateaued in the mature milk. Furthermore, lutein was reported as the dominant nutrient in maternal plasma, cord plasma, transitional milk, and mature milk at up to 400 days postpartum, while beta-carotene was predominant in colostrum. The content of β-carotenoid in middle and late mature breast milk was related to dietary intake (r = 1.690, *p* < 0.05). Carotenoid level in cord blood was lower than that in the mother’s plasma and was related to the carotenoid intake in the mother’s diet. Correlation of carotenoids between maternal and umbilical cord blood, breast milk, and maternal blood could well reflect the transport of carotenoids. These findings may help to guide mothers’ diets during breastfeeding.

## 1. Introduction

Breast milk is considered the best source of nutrition for infants, while exclusive breastfeeding is recommended for 4~6-month-olds. Human breast milk contains adequate nutrients and bioactive substances that babies need in the first six months of life, with carotenoids being one of them [1,2]. Carotenoids are one of the five natural pigment families, which is widely distributed in plants, photosynthetic microorganisms, and some fungi. Carotenoids are also found in other foods such as eggs, corn, oil, and some fish. According to structural characteristics, carotenoids consist of carotene and its hydroxyl derivatives containing only hydrocarbons, including β-carotene, α-carotene and lycopene, and hydroxyl derivatives (e.g., lutein, zeaxanthin, and β-cryptoxanthin) [3,4,5]. Among them, β-carotene, α-carotene, and β-cryptoxanthin can be converted into vitamin A in the body; hence, they are called pro-vitamin A carotenoids, which have certain positive impact on human health, such as enhancing immune functions and promoting visual development [6,7]. Lutein and zeaxanthin mainly exist in the eyes and brain, accounting for 80% to 90% of carotenoids in the human eyes [8]. One of the most significant functions of lutein in the human body is to constitute the pigment of the macular area of the retina [9]. Lycopene may also play a role in immune development and the prevention of inflammatory diseases [10,11].

Carotenoids cannot be synthesized in the human body and can only be obtained through diet [12]. Several studies have investigated carotenoids content in breast milk and found that the content and composition of carotenoids in breast milk changes during lactation [13,14]. For infants who cannot be breastfed, formula milk is an alternative, and infant formula milk commonly refers to breast milk nutrient composition as “gold standard” [15]. However, predicting the absorption of fortified nutrients in infant formula for babies is a challenge, so a few studies have reported that infant serum increase is driven by the intake of lutein from fortified infant formula [16] and breast milk [17]. The correlation of lutein absorption difference in formula and breast milk was also studied. A prospective, double-masked trial showed that carotenoid-fortified infant formula could increase the amount of lutein in serum to the level of infants, although breast-fed infants showed greater linear regression as fourfold that of infant formula [18]. This absorption difference may be associated with the different bioavailability and delivery system of carotenoids between fortified lutein in infant formula and breastmilk.

The fetus and the mother are connected by an umbilical cord, and nutrients pass through the umbilical cord from the mother to the fetus. The correlation varies between the concentration of carotenoids in maternal and cord blood, which could significantly reflect placental carotenoid transport. At present, reports on nutrient levels in maternal/cord plasma and breast milk of Chinese lactating mothers have mostly focused on fatty acids [19,20,21] and macronutrients (e.g., lactose, protein) [22,23]. Only a few studies, specifically long-term longitudinal studies, have focused on carotenoids in breast milk at different stages of lactation. The current study aimed to (1) analyze the trend of changes in the contents of carotenoids (i.e., lutein, zeaxanthin, β-cryptoxanthin, β-carotene, and lycopene) and the corresponding dietary intake during the five stages of lactation in the southern coastal city of Guangzhou, China, to provide additional insights into the importance of carotenoids in fetus development and infants’ health, and (2) investigate the corresponding profiles and contents of carotenoids in maternal and cord plasma.

## 2. Materials and Methods

### 2.1. Participants

Healthy mothers who met the inclusion and exclusion criteria were recruited.

Inclusion criteria: (1) lactating mothers should be between 20 and 35 years old; (2) gestational weeks should be between 37 and 42 weeks; (3) singleton delivery; (4) planned breastfeeding time is 0 to 400 days; (5) newborn should be healthy, with an Apgar score > 8, which is appropriate for gestational age.

Exclusion criteria: (1) nursing mothers suffering from gestational hypertension, gestational diabetes, and other diseases that affect nutrient metabolism; any acute or chronic infectious diseases; and severe heart or kidney disease, and (2) lactating mothers currently taking drugs that affect nutrient metabolism.

In this study, 358 healthy women were recruited from Clifford Hospital, Panyu District, Guangzhou City, Guangdong Province, China, between March 2018 and June 2019. Of this total, only 333 lactating mothers’ milk was included in the analysis of this study. A total of 363 milk samples were collected from 333 healthy lactating mothers: 30 cases of colostrum, 30 cases of transitional milk, 101 cases of mature milk of 40–45 days, 102 cases of mature milk of 200–240 days, and 100 cases of mature milk of 300–400 days.

This study was conducted according to the guidelines provided in the Declaration of Helsinki. All procedures involving human subjects were approved by the Ethics Committee of Clifford Hospital and registered in the China Clinical Trial Center (ChiCTR1800015387) as part of the Maternal Nutrition and Infant Investigation (MUAI) study. Written informed consent was obtained from all participants.

### 2.2. Collection of Basic Demographic Characteristics

Basic information of mothers, including age, education level, occupation, pregnancy and parity, medical history, delivery status, weight gain during pregnancy, pre-pregnancy BMI, and pre-natal BMI, was collected through questionnaires.

### 2.3. Dietary Survey

Dietary intake data of lactating mothers were collected by food frequency questionnaire (FFQ). Before the survey, investigators would be uniformly trained. Under the guidance of the investigators, the lactating mother filled out the designed FFQ questionnaire of dietary intake one month before the sample collection. Investigators would check the questionnaires carefully and contact the lactating mothers for clarification (if necessary). The main concern was the type and amount of carotenoids that lactating mothers take in through their diet. The double entry method was adopted in the questionnaire to avoid mistakes.

### 2.4. Breast Milk and Plasma Sample Collection

Milk of nursing mothers was collected after childbirth, before breakfast and lunch, about 12 o’clock daily, under light protection conditions, Milk of the breast on one side of the lactating mothers was collected in a sterile photo-avoidant centrifuge tube. If one side of the milk could not reach 50 mL, then milk on both sides of the mixture was taken, and 50 mL was placed in the centrifuge tube. The remaining milk can continue to feed the baby. The obtained milk was placed in a refrigerated box and sent to the laboratory within 5 h. After filling, it was stored in a −80 °C refrigerator for testing.

A total of 30 pairs of maternal blood and umbilical cord blood samples (5 mL) were collected using vacutainers (BD vacutainer, lithium heparin blood collection tubes) during parturition. Thereafter, the samples were centrifuged at 1500× *g* centrifugal force for 15 min to gather the plasma within 30 min. Plasma samples were also frozen at −80 °C.

All milk and plasma samples were transported to the Abbott Nutrition Research and Development Centre in Shanghai for analysis within five months of collection.

### 2.5. Determination of Carotenoids Content

#### 2.5.1. Sample Processing

The frozen 50 mL breast milk sample was taken out from the −80 °C refrigerator, thawed at room temperature away from light until it melted, mixed evenly through 2 min vortex or ultrasound for 30 s; then, a clean and sterile 50 mL centrifuge tube was used to transfer 0.5 mL mixed breast milk, and 4 mL pure water and 0.5 g sodium ascorbate were added into the test tube and mixed evenly. Saponification was carried out thereafter. Methanol (10 mL), potassium hydroxide solution (concentration 45%, 3 mL), and tetrahydrofuran (1 mL) were added and mixed evenly, and saponification reaction was carried out. The evenly mixed tube was placed under nitrogen for 5 min to discharge the oxygen in the tube and heated thereafter under a water bath condition of 60 °C for 15 min. After saponification, waiting time was provided until cooling to 37 °C. A total of 10 mL of pure n-hexane and methyl tert butyl ether mixture was added according to the volume ratio of three to one, and vigorous vortex mixing was conducted to extract carotenoids. Thereafter, 15 mL pure water was added again, the mixture was shook well, and then it was centrifuged on the centrifuge at a speed of 2000 rpm for 5 min. The supernatant was separated; 2 mL supernatant was placed into a clean glass tube; the mixture was blow dried with nitrogen again; oxygen was discharged; and the mixture was then redissolved (redissolved in the mixture of methanol and methyl tert butyl ether with a volume ratio of 200 μL to 1), filtered, and left to wait for measurement.

#### 2.5.2. Determination of Carotenoid Levels

All milk samples were analyzed by high-performance liquid chromatograph Agilent 1260 infinity, and the injection amount of each sample was 50 μL. Running time was 55 min, and elution was carried out at a speed of 1 mL/min. Mobile phase A was ammonium acetate aqueous solution, and mobile phase B was acetonitrile/ether/methanol mixed solution (76/9/15, *w*/*w*/*W*); elution gradient was set to 0–20 min, and 75% mobile phase B; 20–22 min, 78% mobile phase B; 22–22.1 min, 80% mobile phase B; 22.1–30 min, 100% mobile phase B; 30–42 min, 100% mobile phase B; 42–42.1 min, 75% mobile phase B; and 42.1–55 min, 75% mobile phase B. Five types of carotenoids were detected by ultraviolet light, and five standard solutions of food carotenoids with different concentrations were prepared thereafter. The corresponding standard curves were drawn for the quantitative analysis of carotenoids in human milk.

### 2.6. Quality Control

Breastmilk samples in Guangzhou were collected, packed, and stored in accordance with standards and uniform procedures. Dietary questionnaires were verified before, and data were entered into the same system. Each breast milk sample was tested in parallel, and the average of the two was taken as the measured value of the breast milk sample. The difference between the two parallel samples must be below 10% of the average of the two, otherwise, the sample needs to be re-measured. Sample pretreatment and detection operations were completed in an environment where ultraviolet rays had been filtered to prevent ultraviolet rays from damaging carotenoids.

### 2.7. Data Processing and Analysis

Data were tested using the Shapiro–Wilk test and non-normally distributed. Thus, categorical data were expressed as percentages, and continuous data were expressed in median and quartile ranges (P25, P75) and (minimum, maximum). The Wilcoxon U test was used to compare the concentration of carotenoids in breast milk during different periods of lactation. Categorical data of demographic characteristics were expressed by the number of cases and percentages. Correlation between the content of carotenoids in breast milk and dietary intake of carotenoids in lactating mothers were tested using Spearman’s correlation. Wilcoxon’s symbolic rank test was used to examine the differences between plasma values in pregnant women and cords. Spearman’s correlation was used to test the correlation between carotenoid levels in maternal and umbilical cord plasma/breast milk. Using SPSS Statistics 26.0 (IBM), *p* < 0.05 was considered statistically significant.

## 3. Results

### 3.1. Sample Size

A total of 363 breastmilk samples were collected in Guangzhou, including 30 cases of postpartum colostrum at 0–5 days, 30 cases of transitional milk at 10–15 days postpartum, 101 cases of mature milk at 40–45 days postpartum, 102 cases of mature milk at 200–240 days postpartum, and 100 cases of mature milk 300–400 days postpartum. Among them, colostrum and transitional milk were provided by the same mother.

A total of 30 pairs of maternal blood and umbilical cord blood samples (5 mL) were collected from mothers who donated their colostrum and transitional milk.

### 3.2. General Demographic Characteristics of Lactating Mothers

Table 1 shows that the ages of lactating mothers in the five lactation stages were around 29 years old, and the average pregnancy weight gain, pre-pregnancy BMI, and prenatal BMI of the lactating women are within the normal range. At the colostrum stage and transitional period, 80.0% of lactating mothers had a college education level or above, and 80% of mothers chose natural delivery methods. In the 40–45 days after childbirth group, 84.2% of lactating mothers had a bachelor’ degree or above, and 55.4% of them chose natural delivery methods. In the 200–240 days postpartum group, 80.4% gave birth naturally, and 82.4% had a bachelor’s degree or above. In the 300–400 days postpartum group, 76% of lactating mothers gave birth by natural delivery, and 77% of them had a bachelor’s degree or above.

### 3.3. Carotenoid Levels and Composition in Maternal and Cord Plasma

Table 2 shows that the concentration of carotenoids in maternal plasma was all markedly higher than that in cord plasma (*p* < 0.001). In maternal plasma, the concentration of β-carotene was the highest among the five carotenoids, followed by lutein, β-cryptoxanthin, lycopene, and zeaxanthin. The concentration of lutein in cord plasma was the highest, followed by β-cryptoxanthin, β-carotene and zeaxanthin, and lycopene. The concentrations of lutein, zeaxanthin, and β-cryptoxanthin in maternal plasma were approximately six to eight times higher than those in cord plasma. In addition, the levels of β-carotene and lycopene were 16–25 times higher than those in cord plasma.

**Figure 1:**
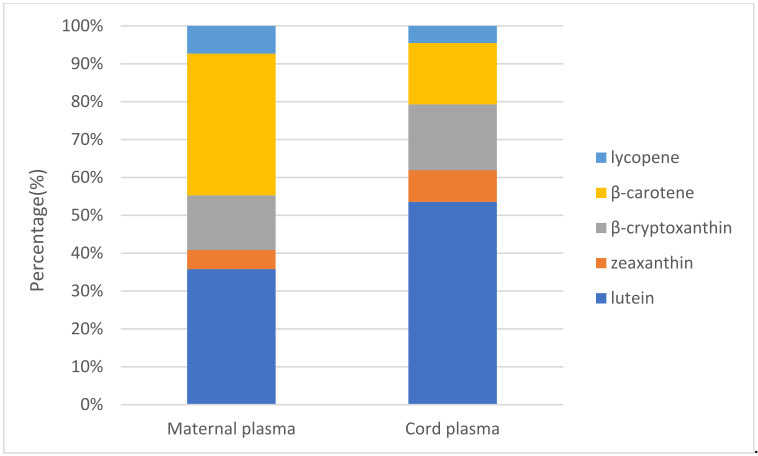
Proportions of carotenoids in plasma (*n* = 30).

Figure 1 shows that the proportions of carotenoids in maternal plasma were as follows: β-carotene, 37%; lutein, 36%; β-cryptoxanthin, 14%; lycopene, 8%; and zeaxanthin, 5%. The proportions of carotenoids in cord plasma were as follows: lutein, 54%; β-cryptoxanthin, 17%; β-carotene, 16%; zeaxanthin, 8%; and lycopene, 5%. Lutein and β-carotene accounted for the most in maternal plasma, while lutein took up the largest portion in cord plasma.

### 3.4. Correlation of Carotenoids Levels in Maternal/Cord Plasma and in Breastmilk

Correlations for all carotenoids between plasma and human milk (*n* = 30) are presented in Table 3 and Table 4. The concentration of each carotenoid in maternal plasma was strongly correlated with that in cord plasma (*p* < 0.001 or *p* < 0.05). For zeaxanthin, β-cryptoxanthin, β-carotene, and lycopene, positive correlations were observed between maternal plasma and colostrum, as well as transitional milk. In lutein, correlations were not significant for maternal plasma and transitional milk.

### 3.5. Trends in Carotenoid Levels in Breast Milk from 0–400 Days Postpartum in Guangzhou

**Figure 2:**
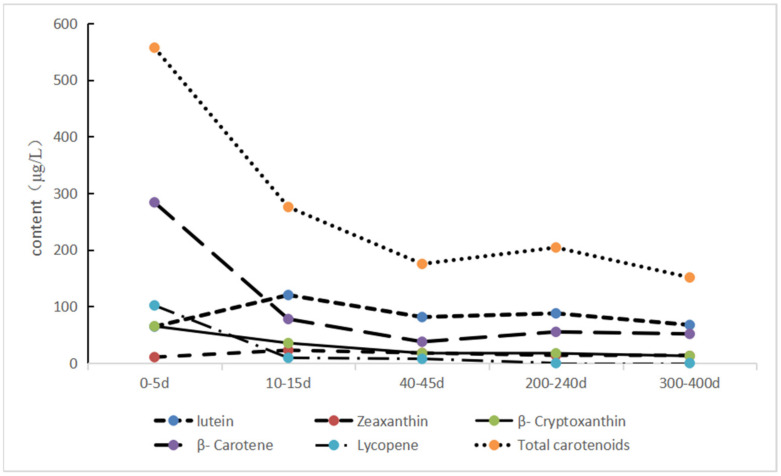
Trends of the content of carotenoid in five lactation stages milk in the Guangzhou area (μg/L).

Table 5 and Figure 2 show that the contents of total carotenoid in colostrum and transitional milk in Guangzhou were higher than that in mature milk (all *p* < 0.05), with medians of 557.25 μg/L and 275.80 μg/L, respectively. However, the content of total carotenoids in three stages of mature milk tended to be relatively stable. Lutein and zeaxanthin showed a trend of initially increasing and decreasing thereafter with the lactation process, showing a stable level in the middle and late mature milk. Moreover, their content in transitional milk was the highest (both *p* < 0.05), being 120.27 μg/L and 22.77 μg/L, respectively. The β-cryptoxanthin in colostrum and transitional milk was higher than that in the middle and late mature milk (both *p* < 0.05), but the content in mature milk did not change significantly. Contents of β-carotene and lycopene in colostrum were higher than those in breast milk in other stages (all *p* < 0.05), and the contents were 283.96 μg/L and 101.98 μg/L, respectively.

### 3.6. Dietary Survey

A total of 436 FFQs were included, among which 427 questionnaires were recycled and 318 valid questionnaires were included in the analysis; the percentage of valid questionnaires was 72.94% (see Table 6 for details).

### 3.7. Dietary Carotenoid Intake Estimated by FFQ

Estimated usual intake of total carotenoids and the five types of carotenoids in five stages of lactating mothers are shown in Table 7. Note that there were differences in the daily intake of total carotenoids and the five carotenoids of lactating mothers at different stages of lactation in the month before the survey (*p* < 0.05). For example, during the 40–45 days of lactation, lactating mothers’ intake of lutein and zeaxanthin differed from that of the other four lactation stages.

### 3.8. Correlation between the Content of Carotenoid in Breast Milk and Estimated Dietary Intake at Various Stages

Table 8 shows that according to the FFQ analysis, no significant correlation was identified between dietary intake of carotenoid (*p* > 0.05) and content of carotenoid in breast milk at the colostrum, transition, and early mature milk stages. However, a positive correlation was identified between the dietary intake of β-carotene and content of β-carotene in middle and late mature milk (r = 1.690, *p* < 0.05), but not for other carotenoids.

## 4. Discussion

### 4.1. Levels of Carotenoids in Breast Milk and Association with Dietary Intake

At present, only a few on longitudinal studies have been conducted on the content of carotenoids in Chinese breast milk and plasma. This study investigated carotenoid levels at different lactating periods and the carotenoid concentrations in maternal and umbilical cord plasma in Southern China. Thus, the result can provide scientific data to understand the content of carotenoid in breast milk. With a longitudinal donor, the potential association of carotenoids among dietary intake, breast milk, and plasma can be further explored.

Our study revealed significant differences in the concentration of carotenoids in breast milk during different lactation periods. We found that the content of carotenoids in maternal plasma was much higher than that in cord plasma in Chinese mothers. In cord plasma, lutein is the dominant nutrient, but not for maternal plasma, in which β-carotene and lutein are both dominant nutrients. This may imply that the potential crucial role of lutein in fetus development and potential different transferring mechanisms of the carotenoids. The concentrations of all carotenoids in maternal plasma were correlated with those of cord plasma as well as human milk (colostrum and transitional milk). Compared with previous studies (*n* = 42 [24], *n* = 56 [25]), as the total content of carotenoids in breast milk continuously decreased over lactating periods [14,26], the current study showed a similar change trend but a higher level at the transitional milk stage and 200–240 days postpartum. For the five carotenoids, except in colostrum, lutein was demonstrated as the predominate component at approximately 45% of the total carotenoids throughout the rest of the lactation stages. Moreover, the lutein level even substantially impacted the change trend of the total carotenoids. This result is aligned with a previous study from MUAI, which also reported the dominant distribution of lutein in transition milk and mature milk (as 51.64% to 52.94% of the total carotenoids), but the study site was in Shanghai [24].

Lutein content in the 40–400 postpartum days ranged from 67.41 to 81.34 ug/L, which was close to the results of Xiu et al. on the content of lutein in mature milk at 42–112 days postpartum in Shanghai, China (69 ug/L) [25]. In addition, Xue et al. [26]. reported lutein levels in Chinese breast milk from the different lactation stages (i.e., 57, 70, 29, and 37 µg/L) were lower than the contents in the mature lactation stage (i.e., 65.54, 120.27, 81.34, and 87.93 µg/L) in the current study. Another survey conducted in Hunan Province found that the average concentrations of lutein in breast milk at 4, 8, and 12 weeks postpartum were 46.41, 57.96, and 62.33 µg/L, respectively [27], thereby showing opposite change trends between Xue et al. and this study. Given that lutein and carotenoids are known to be impacted by dietary intake, the compositional distribution of lutein as of total carotenoids can show the consistency and importance of lutein in breast milk as predominate carotenoids in breast milk. Comparing this study result with the national data, the profile and composition level of lutein were different, owing to dietary intake variance. A multi-center study in Japan, Mexico, and the UK found that the concentrations of lutein and zeaxanthin were different in the three countries, and the concentrations of lutein in Japan was higher than those in the UK and Mexico [28]. A longitudinal study of carotenoids in breast milk from urban populations in China, Mexico, and the USA found that Chinese breast milk had the highest levels of lutein and total carotenoids and the lowest levels of lycopene. In all countries and each lactation period, the top four carotenoids were lutein (114.4 nmol/L), β-carotene (49.4 nmol/L), β-cryptoxanthin (33.8 nmol/L), and lycopene (33.7 nmol/L) [13]. Canfield’s research found that the main carotenoid in breast milk in Chile, China, Japan, and the Philippines is lutein; Canada, alpha-carotene; and Australia, the UK, and the USA, beta-carotene [29]. The possible reason for the difference in the carotenoid contents in breast milk is that the sample collection area of each study is different, and the dietary patterns and eating habits in Guangzhou are different from those in other areas of China. However, note that the results of this study are still comparable to studies conducted in other areas of China. For all carotenoid components, the content of lutein in breast milk is consistently highest and that of lycopene is the lowest, as reported in multiple studies in China.

For lutein, this study did not find a positive correlation between lutein content in breast milk and intake of dietary lutein, which is different from other studies in China [27] and Korea [30]. In addition, other studies have reported that the supplements of lutein increased the concentration of lutein in breast milk and plasma of lactating women, and also the concentration of lutein in plasma of infants; however, lutein supplements did not affect the content of other carotenoids [31]. The results of this study are relatively different from previous studies, which may be caused by different sample sizes, different regions and races, and different breastfeeding periods or seasons. Moreover, another possible reason is that at present, China has no comprehensive database on carotenoid intake. Different dietary survey databases are artificially established, and different types of foods included may lead to different results. Further analysis has shown a more evident correlation between lutein in breast milk and dietary lutein, which may be related to its structural characteristics [3,32]. Lutein has hydroxyl groups at its structural end, and zeaxanthin are isomers of each other. Compared with other carotenoids, lutein and zeaxanthin are more polar and easily transferred to lipoproteins responsible for carotenoid transport, being further transferred and stabilized in the milk globules covered by mammary epithelial cells [13]. A study compared the carotenoids in the diet and serum of males and non-lactating women. Results showed that although the intake of carotenoids in women’s diets was lower, carotenoid levels in women’s serum were significantly higher than that in men, thereby indicating that carotenoid content in breast milk is not only related to dietary carotenoid intake, but may also be related to the bioavailability of carotenoids [33]. Therefore, breastfeeding mothers should properly consume lutein-rich vegetables and fruits, and lutein should also be appropriately added to infant formulas.

For β-carotene and lycopenen, this study found that β-carotene content in mature milk in the middle and late stages was positively correlated with dietary intake. This result aligned with previous studies, which reported that the β-carotene and lycopene contents in breast milk increase [34] when the diet is supplemented with carotene and tomatoes. However, our study did not find a relationship between β-carotene and dietary intake in colostrum and transitional milk, which may have been caused by the saturation of β-carotene in breast milk during the transition period, and dietary supplements will not affect the concentration in breast milk [35]. This observation is in contrast with another study on the nutritional composition of Indonesian mothers’ breast milk, which found a positive correlation between β-carotene contents in breast milk and dietary intake [36].

### 4.2. Levels of Carotenoids in Maternal Plasma and Cord Plasma

We observed that each carotenoid concentration in cord plasma and breast milk was correlated well with that in maternal plasma. This result may indicate that carotenoid concentration in maternal plasma can predict the corresponding carotenoid concentration in newborns. In addition, a correlation was identified between maternal plasma and the two stages of breast milk. Hence, this result showed that carotenoids in mother’s bodies could maintain a stable level within a month after delivery. However, how these higher carotenoid concentrations detected in plasma affect maternal tissue concentrations is not clear, thereby requiring additional in-depth research.

We observed that the content of carotenoid in maternal plasma was considerably higher than that in cord plasma, which is similar to the results of Sun et al. [37]. This can be attributed to a decreased transport capacity for lipophilic substances, such as carotenoids in serum/plasma, owing to low levels of circulating lipoproteins in fetal blood [38].

Previous studies have suggested that carotenoid levels of breastfed infants depend on carotenoid concentration in breast milk, which is affected by mothers’ dietary intake and health status [13]. In addition, infants with lower levels of antioxidant carotenoids in maternal plasma and breast milk may be more prone to adverse health outcomes and growth retardation [39]. Therefore, a worthwhile endeavor is to explore carotenoid levels in the plasma of mothers and infants to possibly imply their health status and nutrition needs.

Given the current lack of well recognized recommendation on ranges of maternal or infant plasma carotenoid concentrations, our results can provide a certain reference value. This study found that the dominant nutrient in maternal blood is β-carotene, while that in cord blood is lutein (median = 623.77 µg/L and 87.37 µg/L, *n* = 30), which is significantly higher than the results of Xu et al. [25] (median = 21.6 µg/dL and 18.2 µg/dL, respectively, *n* = 56) and Hanson et al. [39] (median = 218.3 µg/L and 45 µg/L, respectively, *n* = 99). Moreover, we speculated that compared with other carotenoids, the enrichment of lutein in cord blood and breast milk may imply that babies have considerable demand for it, which is possibly important, particularly for the growth of Chinese infants.

By studying changes in the concentration of carotenoids in mothers’ breast milk over time, as well as its association with maternal blood, cord blood, and dietary intake, future nutritional supplements should be designed to optimize mothers’ dietary intake to increase the carotenoid concentration in breast milk.

### 4.3. Strengths and Limitation

This study spans a long period (i.e., up to over one year), comprising colostrum (0–5 days), transitional milk (10–15 days), early mature milk (40–45 days), mid-term mature milk (200–240 days), and late mature milk (300–400 days). Colostrum and transitional milk are provided by the same mother, with matched mother’s blood at birth and cord blood. Samples could relatively reflect the characteristics of carotenoid in breast milk during lactation in coastal cities in Southern China. Furthermore, we collected all milk after mixing all breast milk from one side of breast milk of lactating mothers to reduce the difference in carotenoid content in breast milk caused by changes in the composition of the fore, middle, and back milk.

This study also has certain limitations. For example, there was no difference in the content of carotenoid between early mature milk and mid-late mature milk, which may have been caused by the different seasons of sample collection, given that seasonal dietary intakes can be different. Food composition data of carotenoids of this study were established with partial reference to the U.S. food composition data. Consequently, some foods in the survey with Chinese characteristics cannot be covered by the database, thereby jeopardizing the establishment of correlation. Therefore, the database of carotenoid contents in foods in China should be further determined and improved.

## 5. Conclusions

Our study reported the concentrations of five carotenoids in maternal plasma, cord plasma, and human milk over five different lactation stages and their dietary intake of carotenoids in a city of China. We found that with the extension of lactation period, the content of carotenoid in breast milk gradually decreased and reached a stable level. Meanwhile, carotenoid content in maternal plasma was higher than that in cord plasma. The content of carotenoids in cord blood is related to maternal dietary intake and carotenoid levels in maternal blood. We also found that lutein is the dominant nutrient in maternal plasma, cord plasma, transitional milk, and mature milk as up to 400 days postpartum. Whether or not the high expression of lutein in breast milk and plasma reveals the importance of lutein for infant growth and development requires further research. This study provided valuable data in the database of carotenoid contents in breast milk, maternal plasma, and cord plasma in China, and contributed to the development of dietary recommendations for lactating mothers. Research on carotenoid content in breast milk remains in its infancy, and additional exploratory studies are needed to clarify the correlation between carotenoid content in breast milk and diet, as well as the possible roles in fetus development and infants’ health.

## Figures and Tables

**Table 1:** Basic situation of lactating mothers.

Basic Situation		Colostrum and Transitional Milk (N = 30)	Mature Breast Milk 40–45 Day (N = 101)	Mature Breast Milk 200–240 Day (N = 102)	Mature Breast Milk 300–400 Day (N = 100)
Age		28.50 ±3.31	29.40 ±3.60	30.18 ±3.75	30.28 ±4.05
Weight gain during pregnancy		13.85 ±3.61	13.99 ±4.82	12.81 ±4.71	13.44 ±5.04
BMI before pregnancy		20.64 ±3.75	21.60 ±3.18	20.29 ±2.36	20.38 ±3.05
Prenatal BMI		26.07 ±3.34	26.97 ±3.29	25.36 ±2.96	25.64 ±3.56
Education level					
	Junior high school or below	0 (0%)	7 (6.9%)	4 (3.9%)	4 (4%)
	High school/vocational high school/technical secondary school	6 (20.0%)	9 (8.9%)	14 (13.7%)	19 (19%)
	Undergraduate/college	23 (76.7%)	75 (74.3%)	79 (77.5%)	74 (74%)
	Master’s degree and above	1 (3.3%)	10 (9.9%)	5 (4.9%)	3 (3%)
Delivery method					
	Natural childbirth	24 (80%)	56 (55.4%)	82 (80.4%)	76 (76%)
	Cesarean section	6 (20%)	45 (44.6%)	20 (19.6%)	24 (24%)

**Table 2:** Carotenoid levels (μg/L) in plasma (*n* = 30).

		Maternal Plasma	Cord Plasma	*p* *
Lutein	median	623.77	87.37	<0.001
	P25, P75	455.17, 623.77	67.24, 87.37	
	min, max	268.36, 1495.48	47.49, 194.73	
Zeaxanthin	median	88.30	13.68	<0.001
	P25, P75	69.87, 104.89	10.62, 18.46	
	min, max	43.59, 276.31	6.46, 31.15	
β-Cryptoxanthin	median	251.55	28.38	<0.001
	P25, P75	169.34, 654.90	14.99, 49.99	
	min, max	69.46, 1818.91	9.56, 105.20	
β-Carotene	median	652.66	26.47	<0.001
	P25, P75	352.35, 893.52	14.53, 36.64	
	min, max	142.22, 1813.41	4.69, 155.96	
Lycopene	median	127.85	7.37	<0.001
	P25, P75	80.55, 209.85	6.16, 8.49	
	min, max	15.66, 498.14	3.04, 23.21	

* Differences between carotenoid levels in maternal plasma and cord plasma were tested using the Wilcoxon signed-rank test.

**Table 3:** Correlations of carotenoid levels in maternal plasma and cord plasma (*n* = 30).

Carotenoids	Correlation	*p*
Lutein	0.782 **	<0.001
Zeaxanthin	0.749 **	<0.001
β-Cryptoxanthin	0.918 **	<0.001
β-Carotene	0.889 **	<0.001
Lycopene	0.403 *	<0.05

Spearman’s correlation was performed to analyze the correlations between carotenoid levels in maternal plasma and cord plasma. ** *p* < 0.001, * *p* < 0.05.

**Table 4:** Correlations of carotenoid levels in maternal plasma and human milk (*n* = 30).

	Lutein	Zeaxanthin	β-Cryptoxanthin	β-Carotene	Lycopene
Colostrum	0.782 **	0.370 *	0.706 **	0.691 **	0.402 *
*p*	<0.001	0.044	<0.001	<0.001	0.027
Transitional milk	0.220	0.371 *	0.818 **	0.795 **	0.449 *
*p*	0.244	0.044	<0.001	<0.001	0.013

* Correlations were significant at *p* < 0.05, two-tailed; ** Correlations were significant at *p* < 0.001, two-tailed. Spearman’s correlation was performed to analyze the correlations between carotenoid levels in maternal plasma and milk.

**Table 5:** Comparison of the contents of carotenoids in breast milk at five lactation stages in Guangzhou (μg/L, median (P25, P75), (min, max)).

Carotenoids		Colostrum	Transitional Milk	Mature Breast Milk	Mature Breast Milk	Mature Breast Milk	*p*
		(0–5 d)	(10–15 d)	(40–45 d)	(200–240 d)	(300–400 d)	
Lutein	median	64.54 ^a^	120.27 ^b^	81.34 ^a^	87.93 ^a^	67.41 ^a^	<0.001 *
	(P25,	(39.36,	(96.69,	(61.28,	(65.86,	(40.96,	
	P75)	130.57)	173.97)	122.61)	124.69)	106.39)	
	(min,	(21.69,	(48.12,	(10.66,	(12.43,	(8.59,	
	max)	329.95)	374.83)	302.55)	332.24)	209.10)	
Zeaxanthin	median	10.53 ^a^	22.72 ^b^	17.88 ^b^	13.76 ^a^	12.63 ^a^	<0.001 *
	(P25,	(6.69,	(15.72,	(11.41,	(9.22,	(8.11,	
	P75)	15.82)	36.22)	24.55)	19.57)	18.20)	
	(min,	(4.71,	(6.99,	(4.86,	(0,	(5.06,	
	max)	38.23)	76.46)	62.66)	38.76)	69.67)	
β-Cryptoxanthin	median	65.19 ^a^	35.39 ^a^	17.55 ^b^	17.33 ^b^	12.66 ^b^	<0.001 *
	(P25,	(33.71,	(21.82,	(10.74,	(12.12,	(7.78,	
	P75)	118.22)	76.07)	28.12)	23.97)	20.95)	
	(min,	(9.28,	(6.57,	(2.88,	(0,	(4.08,	
	max)	205.71)	138.72)	134.83)	174.56)	122.98)	
β-Carotene	median	283.96 ^a^	77.84 ^c^	37.71 ^c^	55.19 ^b^	51.63 ^b^	<0.001 *
	(P25,m	(153.68,	(45.45,	(23.41,	(30.20,	(29.62,	
	P75)	472.12)	120.29)	66.49)	84.77)	83.37)	
	(min,	(71.77,	(19.48,	(4.19,	(3.71,	(3.76,	
	max)	1905.48)	422.19)	208.42)	375.2)	232.55)	
Lycopene	median	101.98 ^a^	9.34 ^b^	7.59 ^b^	0	0	<0.001 *
	(P25,	(74.13,	(6.69,	(4.06,	(0,	(0,	
	P75)	227.09)	17.49)	11.38)	2.66)	5.42)	
	(min,	(24.31,	(1.39,	(0,	(0,	(0,	
	max)	524.58)	35.45)	48.38)	25.98)	29.52)	
Total carotenoids	median	557.25 ^a^	275.80 ^a^	175.24 ^b^	204.26 ^b^	151.33 ^b^	<0.001 *
	(P25,	(351.64,	(231.72,	(132.27,	(138.67,	(93.64,	
	P75)	930.56)	372.29)	245.39)	262.41)	255.65)	
	(min,	(133,	(94.40,	(46.51,	(30.54,	(35.64,	
	max)	3003.90)	829.40)	468.61)	801.79)	551.31)	

Data are expressed in median (P25, P75), (min, max); ^a,b,c^ different footnote letters in the same line represent statistical differences (* *p* < 0.001).

**Table 6:** The recovery of valid questionnaires in Guangzhou (unit: copies).

Questionnaire Type	Colostrum	Transitional Milk	40–45 d	200–240 d	300–400 d	Total
FFQ	30	30	67	97	94	318

**Table 7:** The dietary intake and comparison of carotenoids in different stages of lactation (μg/L).

Carotenoids		0–5 Day	10–15 Day	40–45 Day	200–240 Day	300–400 Day	*p*
Lutein + zeaxanthin	median	1166.54 ^b^	1833.72 ^b^	1176.93 ^a^	1581.69	1050.19 ^b^	<0.05
	(P25,	(603.86,	(1229.06,	(538.95,	(876.10,	(603.51,	
	P75)	1819.34)	3596.69)	1728.54)	3280.11)	2337.19)	
	(min,	(170.31,	(181.44,	(133.36,	(0,	(177.86,	
	max)	5136.53)	7501.13)	6071.07)	19,398.30)	38143.99)	
β-Cryptoxanthin	median	69.32 ^a^	61.64 ^b^	57.71	59.2	59.37	<0.05
	(P25,	(34.51,	(48.78,	(14.81,	(14.3,	(18.09,	
	P75)	139.92)	139.01)	114.07)	126.42)	124.38)	
	(min,	(34.51,	(3.87,	(1.29,	(0,	(0.93,	
	max)	658.60)	1048.64)	411.93)	711.07)	768.48)	
β-Carotene	median	665.44	1141.31 ^a^	416.61 ^b^	772.61 ^b^	520.51 ^c^	<0.05
	(P25,	(530.81,	(604.23,	(230.58,	(381.65,	(287.95,	
	P75)	1708.15)	2017.69)	1083.43)	2274.31)	1155.50)	
	(min,	(55.89,	(36.52,	(11.97,	(0,	(27.27,	
	max)	4602.61)	3845.22)	3241.41)	11,853.36)	18,592.52)	
Lycopene	median	1194.73 ^a^	229.73 ^c^	275.68 ^ac^	875.29 ^a^	842.18 ^a^	<0.05
	(P25,	(470.37,	(180.72,	(0,	(367.57,	(183.94,	
	P75)	2481.35)	787.30)	1102.71)	1929.75)	2045.57)	
	(min,	(0,	(0,	(0,	(0,	(0,	
	max)	11,697.57)	5146)	5810.14)	7748)	13,981.95)	
Total carotenoids	median	3280.68 ^b^	5419.57 ^b^	3296.59	5822.48 ^a^	4184.62 ^b^	<0.05
	(P25,	(2106.34,	(4058.79,	(1959.31,	(3190.88,	(2187.08,	
	P75)	5180.03)	6472.54)	6114.29)	10,309.95)	6651.63)	
	(min,	(498.69,	(377.92,	(6.43,	(0,	(532.24,	
	max)	9443.48)	10,362.01)	23,412.86)	43,273.03)	35,014.78)	

Data are expressed as median (P25, P75), (min, max); ^a,b,c^ different footnote letters in the same line represent statistical differences (*p* < 0.05).

**Table 8:** Association between dietary intake and the content of carotenoid in breast milk at postpartum 0–400 days.

	β-Carotene	β-Cryptoxanthin	Lycopene	Lutein + Zeaxanthin	Total Carotenoids
colostrum	0.184	0.012	−0.237	0.052	−0.041
*p*	0.332	0.951	0.207	0.786	0.829
Transitional milk	0.296	0.345	0.114	0.239	−0.274
*p*	0.113	0.062	0.548	0.203	0.143
Early mature milk	0.189	−0.004	0.094	−0.037	−0.052
*p*	0.640	0.971	0.358	0.722	0.614
Middle and late mature milk	1.690 *	0.058	−0.008	−0.033	−0.015
*p*	0.016	0.416	0.907	0.639	0.836

* *p* < 0.05.

## Data Availability

No new data were created or analyzed in this study. Data sharing is not applicable to this article.
